# Efficacy and safety of cyclin‐dependent kinase 4/6 inhibitor in patients with advanced breast cancer: A real‐world experience

**DOI:** 10.1111/1759-7714.15090

**Published:** 2023-09-04

**Authors:** Tzu‐Rong Peng, Jia‐Hui Chen, Ta‐Wei Wu

**Affiliations:** ^1^ Department of Pharmacy, Taipei Tzu Chi Hospital Buddhist Tzu Chi Medical Foundation New Taipei City Taiwan; ^2^ Division of General Surgery, Department of Surgery, Taipei Tzu Chi Hospital The Buddhist Medical Foundation New Taipei City Taiwan; ^3^ School of Medicine Buddhist Tzu Chi University Hualien Taiwan

**Keywords:** cyclin‐dependent kinase inhibitor, advanced breast cancer, efficacy, safety

## Abstract

**Background:**

Cyclin‐dependent kinases 4 and 6 inhibitors (CDK4/6i) have been shown to improve progression‐free survival (PFS) in patients with metastatic breast cancer (MBC) in randomized control trials. This study aimed to evaluate the efficacy and safety of CDK4/6i in patients with advanced breast cancer (ABC) in a clinical setting.

**Methods:**

Consecutive patients with ABC were treated between October 2019 and March 2023 at Taipei Tzu Chi Hospital, Taiwan. Patients who had received at least one dose of CDK4/6i were included in this retrospective study. The main outcome of this study was efficacy based on the treating physicians' assessments in terms of PFS, and overall survival (OS), as well as the factors associated with patient outcome. The secondary outcome was safety.

**Results:**

A total of 85 patients were included in the analysis, with a mean age of 66.8 years. After a median follow‐up of 16.1 months, the median PFS was 28.4 months (95% CI: 22.5–33.6) and the median OS could not yet be estimated. The most common adverse events (AE) were fatigue (50.8%), anorexia (45.9%), and leukopenia (44.7%). In multivariable analysis, treatment with CDK4/6i with any grade AE or response to treatment effect (CR/PR) was an independent predictor for longer PFS (hazard ratio [HR] = 0.27, 95% CI: 0.11–0.68; HR = 0.21, 95% CI: 0.06–0.67; *p* < 0.05).

**Conclusion:**

CDK4/6i administered in a real‐world setting exhibits a similar survival benefit with the clinical trials.

## Graphical abstract

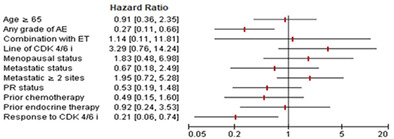

## INTRODUCTION

Breast cancer is the most common cancer in women and accounts for the leading cancer mortality in women worldwide. The subtype of breast cancer has emerged as an important strategy for guidance in optimal treatment.^1^ Hormone receptor‐positive (HR+) and human epidermal growth factor receptor 2 negative (HER2−) breast cancer represent one of the most common subtype diagnoses among women.^2^, ^3^ Endocrine therapy (ET) is the standard intervention for the treatment of HR+/ HER2− ABC. ET (e.g., letrozole or fulvestrant) can delay the progression of metastatic disease; however, most patients finally progress through treatment.^2^ The development of resistance to ET through diverse mechanisms.^4^

Dysregulation in the cyclin D–CDK–retinoblastoma pathway is usually the reason for resistance to endocrine monotherapy, therefore, making CDK 4/6 a highly relevant target for endocrine resistance in patients with ABC. Many studies have shown that combined use of CDK4/6i and ET can successfully prolong progression‐free survival (PFS) and improve overall survival (OS) compared with ET alone.^5^, ^6^, ^7^, ^8^ At the same time, randomized trials have shown that CDK4/6i combined with ET may lead to dose reduction or treatment interruption due to drug‐related adverse events (AEs) (such as neutropenia).^9^ However, the overall risk for infection is low and these agents are generally considered to be well‐tolerated, despite clinically significant AEs such as QT interval prolongation mainly due to ribociclib.^10^ The purpose of this study was to evaluate the efficacy and safety of CDK4/6i in therapy for ABC in a real‐world setting.

## METHODS

### Ethics statement

This study was conducted according to the principles of the Declaration of Helsinki and was approved by the Ethical Committee of Taipei Tzu Chi Hospital, Buddhist Tzu Chi Medical Foundation. Informed written consent was waived because the study was a retrospective data analysis.

### Study design, data sources, and study population

This was a retrospective study whose objective was to evaluate the safety and efficacy of CDK4/6i in the treatment of ABC in a real‐world setting. Consecutive patients with metastatic or locally advanced and unresectable breast cancer were treated between October 2019 and March 2023 at Taipei Tzu Chi Hospital, New Taipei city, Taiwan. Eligible patients were pre‐ and postmenopausal women with a histologically proven HR+ ABC, candidates to receive CDK4/6i plus ET as a first or subsequent line of therapy according to their contingent clinical situation. Data collection started from the administration of the first dose of palbociclib or ribociclib and included patients' performance status and age at the study entry, disease characteristics, hormone receptor and HER2 status, sites, and several metastases and tumor biology, as well as previous therapies received in the neoadjuvant, adjuvant, and metastatic setting.

### Main outcome measurements

The main outcomes of this study were OS and PFS. OS was defined as the time from CDK4/6i administration to any‐cause death, the date of loss to clinical follow‐up, or the date of data cutoff. Patients who were lost to follow‐up were censored at the last date the patient was known to be alive, and patients who remained alive were censored at the time of data cutoff (June 30, 2023). PFS was defined as the time from CDK4/6i administration to the first documented date of tumor progression or death, whichever occurred first.

### Treatment response measurements

The response to treatment was defined as the most effective response during the treatment period. Complete response (CR) was defined as the disappearance of any intratumoral arterial enhancement in all target lesions, and partial response (PR) was defined as at least a 30% decrease in the sum of the diameters of the viable target lesions (enhancement in the arterial phase), progressive disease (PD) was defined as at least a 20% increase in the sum of the diameters of the viable target lesions, and stable disease (SD) was defined as neither PR nor PD.^11^

We followed the NCCN guidelines to structure the assessment and evaluation processes when monitoring patients with ABC. The follow‐up involved a combination of clinical evaluations, tumor marker assessments, and imaging studies. The frequency of these assessments varied depending on individual patient factors, disease stage, treatment response, and overall health status. Below are the general guidelines for the frequency of follow‐up tests.

#### Clinical evaluations

*Physical examination*: Regular physical examinations are essential to monitor the patient's overall health and assess any changes in clinical symptoms or disease status and should be provided during each visit.

*Symptom assessment*: Patients should communicate any new or worsening symptoms to our team promptly, and these symptoms should be assessed during each visit.

#### Tumor marker assessment

CA 15–3 is commonly measured in patients with ABC. The frequency of tumor marker assessment may vary but is often done every 3 months or as indicated by the patient's clinical condition.

#### Imaging studies

A chest computed tomography (CT) scan may be performed every 3 to 6 months, or as clinically indicated, to evaluate the chest area for any new or progressing lesions. An abdominal CT scan or sonography may be done every 3 to 6 months or as needed to assess the abdomen for any disease progression or new lesions. Whole‐body positron emission tomography–computed tomography (PET‐CT) scan may be recommended every 6–12 months to provide a comprehensive assessment of disease activity and metastases. Bone scan may be considered every 6–12 months or as clinically indicated to detect any bone metastases or evaluate the response to treatment.

By utilizing established criteria such as RECIST^12^ and WHO,^13^ more accurate and objective assessments of disease activity and treatment response can be made. The objective response rate (ORR) was the proportion of patients who achieved the best overall response of confirmed CR or PR and the disease control rate (DCR) was defined as an ORR or SD.

#### Safety evaluation

Safety was assessed on day 1 of every cycle during treatment and for up to 30 days after the last dose. AE was assessed by the investigator as either related or not related to the study drug. The severity of AE was classified and evaluated according to the National Cancer Institute Common Terminology Criteria for Adverse Events (CTCAE), version 5.0.^14^

### Statistical analysis

Statistical analysis was performed using SPSS version 23.0 (IBM). The baseline characteristics were descriptive statistics and/or frequency tables. Continuous variables are summarized with descriptive statistics (n, mean, and range). Time‐to‐endpoint events (OS and PFS) were analyzed using the Kaplan–Meier method, and the comparisons were computed with the log‐rank test. The association between prognostic factors and survival was examined using Cox proportional hazards regression model. Clinical variables that were included in the models as covariates were age, the best response to CDK4/6i, combination with ET and CDK4/6i, lines for CDK4/6i, prior chemotherapy, prior ET, site of metastasis, metastatic status, and progesterone receptor status. A two‐sided *p*‐value of <0.05 was considered statistically significant.

## RESULTS

### Demographic characteristics of study patients

**FIGURE 1:**
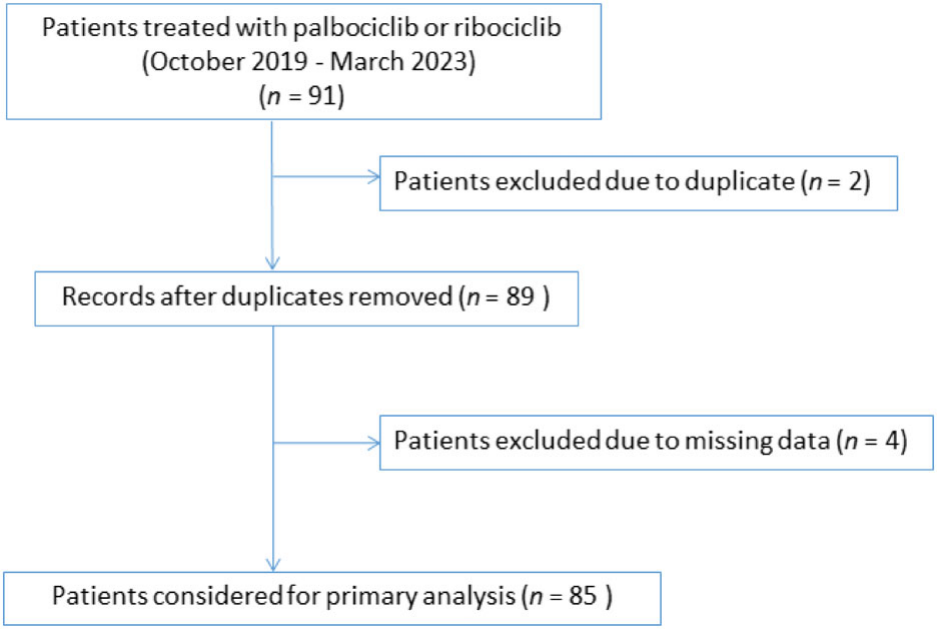
Patient selection flow chart.

During the study period (October 2019 to March 2023), 85 patients with HR+/HER2− ABC, most of them women, were treated at the study hospital. The mean age was 66.8 years (range 27–95 years). A total of 74 patients were postmenopausal and 11 patients were premenopausal. Patients were followed‐up until June 30, 2023. Median follow‐up was 16.1 months (95% CI: 15.5–21.0). A total of 50 patients (58.8%) were treated with ribociclib and 33 (38.8%) with palbociclib. However, two of them used ribociclib and palbociclib, respectively. The mean CDK4/6i treatment duration was 13.4 months. The drug was prescribed as first‐line therapy for 50 patients (58.8%) and as a second‐line or later treatment for 35 patients (41.2%). At the beginning of systemic treatment with CDK4/6i, approximately 78.8% of patients had MBC. A total of 33 patients (38.8%) had ≥2 disease sites. CDK4/6i was started on a reduced dose in 21 patients (24.7%), and 64 patients (75.3%) started on the full recommended dose. The most common ET combined with CDK4/6i was letrozole (62 patients; 72.9%), followed was fulvestrant (11 patients; 12.9%). The patient selection flow chart is reported in Figure 1. Clinical and demographic characteristics are presented in Table 1.

**TABLE 1:** Patient demographics and clinicopathological characteristics.

Characteristics	Patients, *N* = 85 (%)
CDK4/6i	
Ribociclib	50 (58.8)
Palbociclib	33 (38.8)
Mixed	2 (2.4)
Age (mean ± SD)	66.8 ± 12.2
<65 years	36 (42.4)
≥65 years	49 (57.6)
Sex	
Male	1 (1.2)
Female	84 (98.8)
Menopausal status	
Premenopausal	11 (12.9)
Postmenopausal	74 (87.1)
Estrogen receptor at the primary disease	
Positive	83 (97.6)
Negative	2 (2.4)
Progesterone receptor at the primary disease	
Positive	67 (78.8)
Negative	18 (21.2)
HER2 at primary disease at the primary disease	
Positive	10 (11.8)
Negative	75 (88.2)
Neoadjuvant and/or adjuvant chemotherapy	
Anthracycline and taxane	4 (4.7)
Anthracycline only	2 (2.4)
Taxane	6 (7.1)
Other	7 (8.2)
None	66 (77.6)
Adjuvant ET	
Aromatase inhibitors	68 (80.0)
Fulvestrant	11 (12.9)
Both	2 (2.4)
None	4 (4.7)
Type of relapse	
Local/locoregional unresectable	18 (21.2)
Metastatic	67 (78.8)
Line of treatment	
First‐line	50 (58.8)
Second‐line	35 (41.2)
Chemotherapy before CDK4/6i	
Yes	19 (22.4)
No	66 (77.6)
ET before CDK4/6i	
Yes	46 (54.1)
No	39 (45.9)

### Treatment efficacy

OS outcomes were not mature at the time of the data cutoff, with 20 deaths (23.5%) in this period. The one‐year OS rate was 96.5%. The median PFS was 28.4 months (95% CI: 22.5–33.6). Seven patients did not have evaluable data for radiological response at the time of the data cutoff. Of the 85 patients, 19 patients achieved CR and 45 had a PR to treatment, an ORR of 75.3% according to routine clinical evaluation, tumor marker, and radiology reports. Furthermore, nine patients had SD as the best response while five patients had disease progression (Table 2).

**TABLE 2:** Treatment response rate.

Characteristics	Patients (*n* = 85)	
	*n*	%
Overall response rate (ORR)	64	75.3
Disease control rate (DCR)	73	85.9
Complete response (CR)	19	22.4
Partial response (PR)	45	52.9
Stable disease (SD)	9	10.6
Disease progression (PD)	5	5.9

**FIGURE 2:**
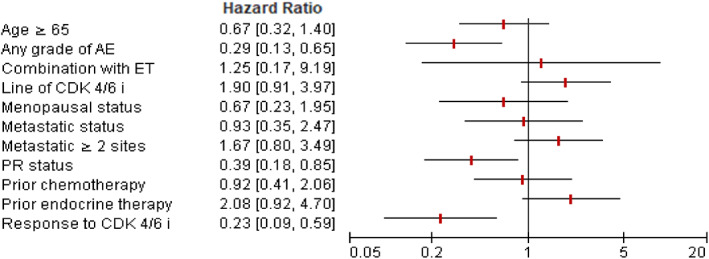
Forest plot of hazard ratio for progression‐free survival, Cox regression univariate model.

**FIGURE 3:**
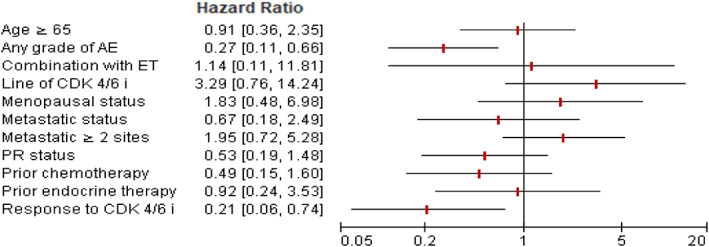
Forest plot of hazard ratio for progression‐free survival, Cox regression multivariable model.

**FIGURE 4:**
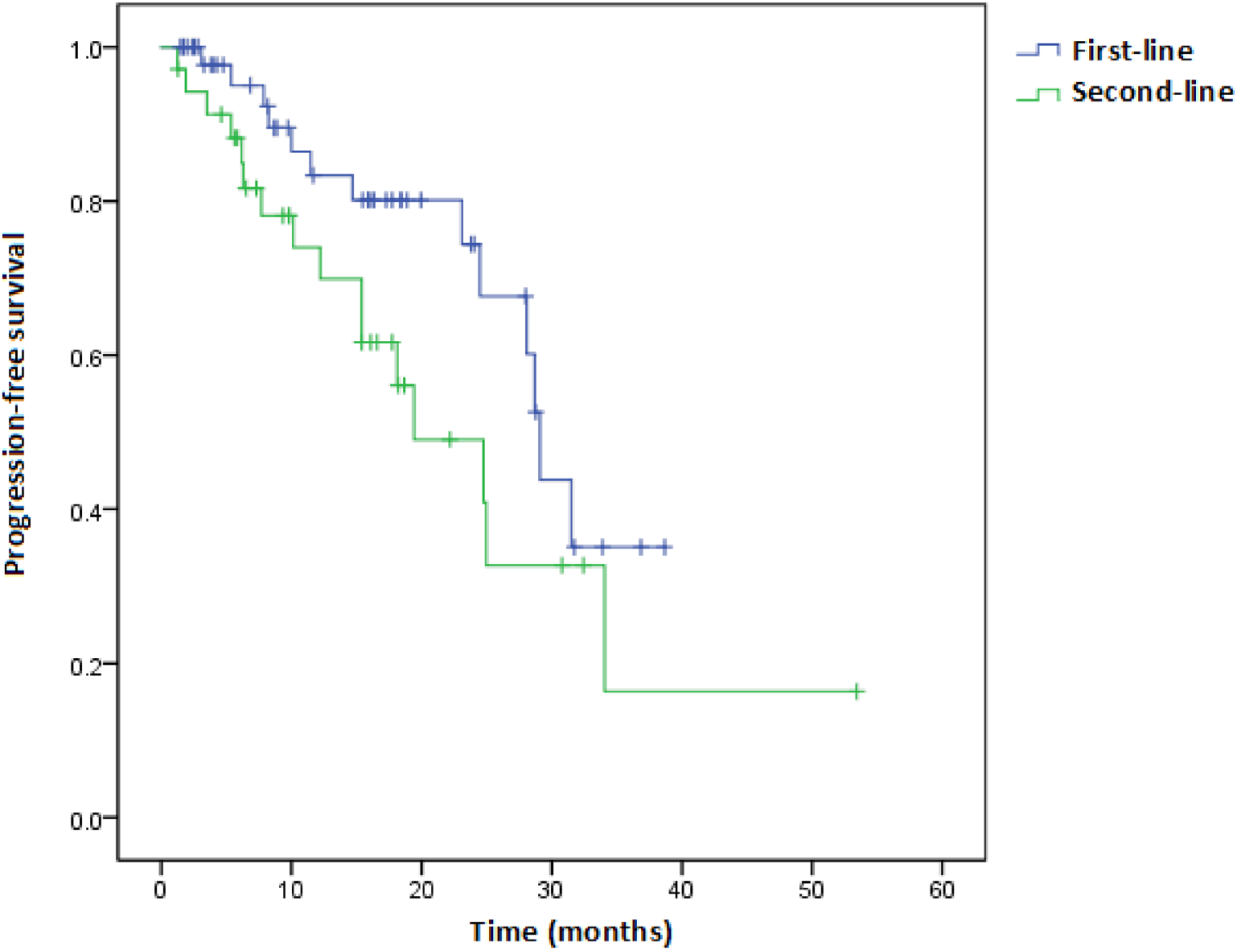
Progression‐free survival in first‐line versus second‐line of CDK 4/6i (log‐rank test, *p* = 0.081).

**FIGURE 5:**
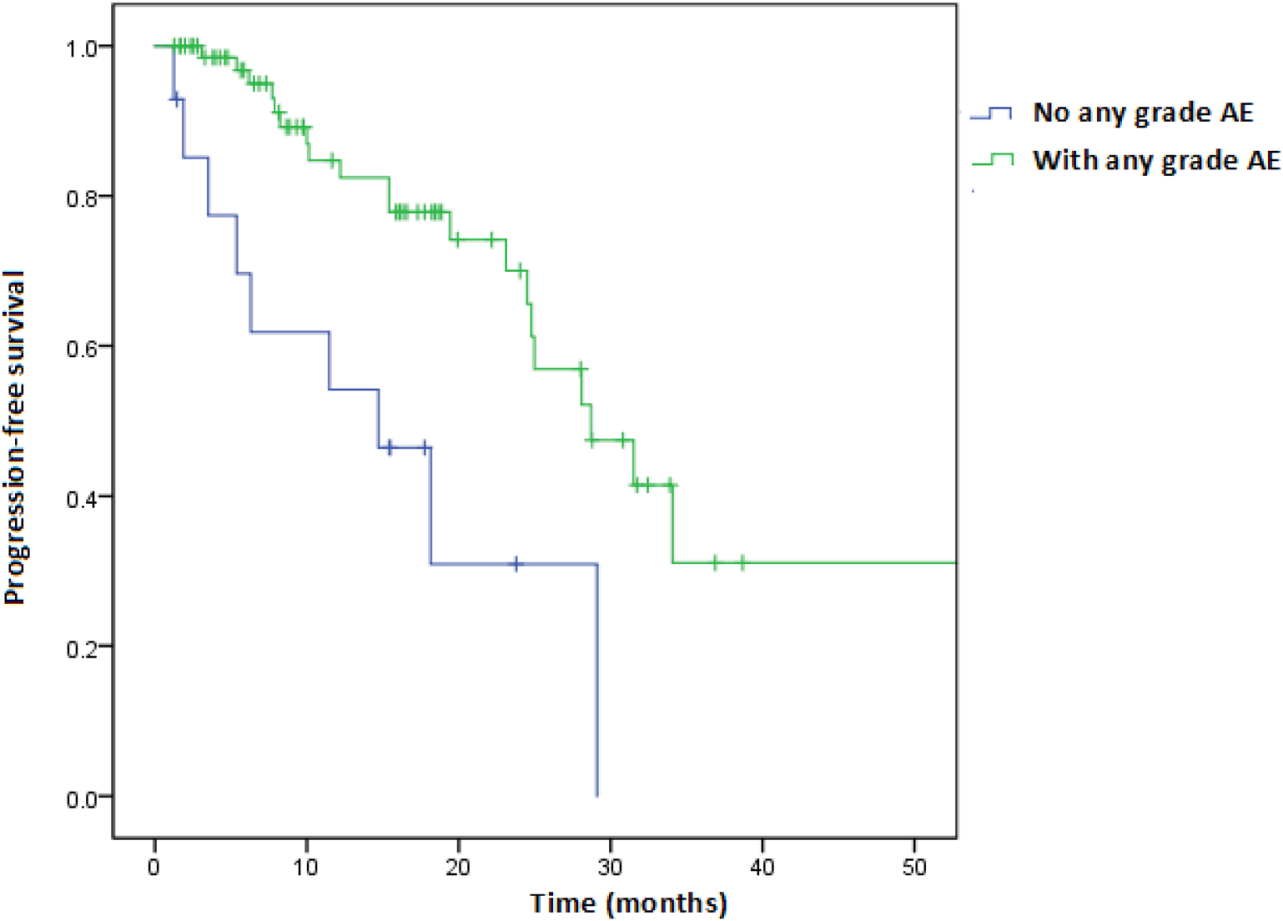
Progression‐free survival in no grade AE versus with any grade adverse event (AE) (log‐rank test, *p* = 0.001).

**FIGURE 6:**
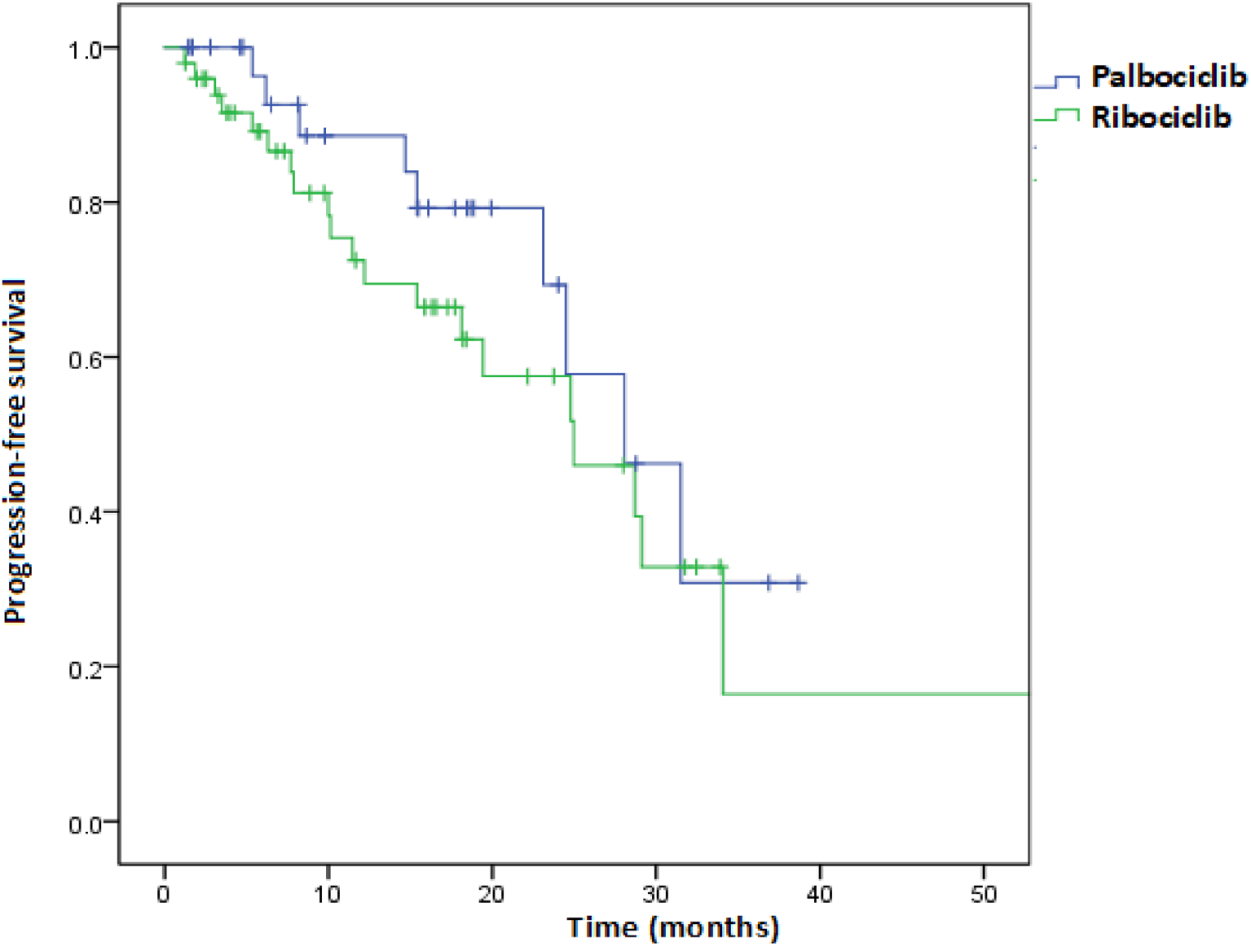
Progression‐free survival in palbociclib versus ribociclib (log‐rank test, *p* = 0.274).

Univariate and multivariable Cox regression analyses of factors associated with PFS are shown in Figures 2 and 3, respectively. Treatment with CDK4/6i with any grade AE or response to treatment effect (CR/PR) was an independent predictor for longer PFS (HR = 0.27, 95% CI: 0.11–0.68; HR = 0.21, 95% CI: 0.06–0.67), when adjusted age, progesterone receptor status, combination with ET, line of CDK4/6i, menopausal status, metastatic status, prior chemotherapy or ET. Furthermore, we investigated whether the line of therapy in which CDK4/6i was administered had an impact on PFS. The median PFS of CDK4/6i in first‐line treatment was 29.1 months (27.5–30.7) compared to 19.4 months (10.1–28.8) for second‐line therapy (log‐rank test *p* = 0.081) (Figure 4). In addition, we assessed a median PFS of 28.7 months (20.6–36.8) for those who have any grade AE in the period of CDK4/6I, whereas it was 14.7 months (4.3–25.1) for patients with no grade AE (log‐rank test, *p* = 0.001) (Figure 5). In general, palbociclib had no significantly higher median PFS with 28.1 months (95% CI: 20.2–35.9) versus 25.0 months ribociclib (95% CI: 14.7–35.2) (log‐rank test *p* = 0.274) (Figure 6).

### Treatment safety

Adverse events of any grade are described for 71 patients (83.5%). Fatigue, anorexia, and leukopenia were the most common AE. The reported incidence rate of any grade fatigue, anorexia, and leukopenia was 50.8%, 45.9%, and 44.7% in patients treated with CDK4/6i. The most common grade 3/4 AE of interest was neutropenia (6 patients; 7.1%), followed by thrombocytopenia (4 patients; 4.7%) and anemia (4 patients; 4.7%). Prolonged QTc time occurred in six cases in patients receiving ribociclib. Four out of six patients had dose reduction as a result. In addition, at least one dose reduction was performed in 23 patients (27.1%) after a median of two cycles (range, 1–18), most commonly due to hematological toxicity (56.5% of cases), followed by fatigue, abnormal liver function, and QT interval prolongs (8.7% of cases) (Table 3).

**TABLE 3:** Adverse events related to ribociclib and palbociclib treatment.

Adverse events (AE)	G1 (%)	G2 (%)	G3 (%)	Any grade
Leukopenia	6 (7.1)	26 (30.6)	6 (7.1)	38 (44.7)
Anemia	11 (12.9)	13 (15.3)	4 (4.7)	27 (29.4)
Thrombocytopenia	2 (2.4)	2 (2.4)	4 (4.7)	8 (9.4)
Anorexia	25 (29.4)	14 (16.5)	0 (0)	39 (45.9)
Nausea	32 (37.6)	1 (1.2)	0 (0)	33 (38.8)
Skin rash	25 (29.4)	3 (3.5)	0 (0)	28 (32.9)
Alopecia	24 (28.2)	3 (3.5)	0 (0)	27 (31.8)
Fatigue	46 (54.1)	4 (4.7)	0 (0)	50 (58.8)
Hand‐foot syndrome	30 (35.3)	1 (1.2)	0 (0)	31 (36.5)
Abnormal liver function	2 (2.4)	2 (2.4)	1 (1.2)	5 (5.9)
Abnormal kidney function	3 (3.5)	0 (0)	0 (0)	3 (3.5)

## DISCUSSION

Cyclin‐dependent kinases 4 and 6 (CDK 4/6) are cell‐cycle regulators that combine with cyclin D to hyperphosphorylate retinoblastoma (Rb), inactivating and uncoupling G1‐ to S‐phase cell‐cycle progression. The uncontrolled formation of cyclin D1 and CDK 4/6 complexes plays an integral role in both the initiation and progression of breast cancer and may be associated with endocrine resistance.^15^, ^16^ The CDK4/6i combined with ET has proven to be PFS in women with HR+/HER2− MBC. Two randomized clinical trials (PALOMA and MONALEESA studies) have demonstrated that they are safe and well tolerated and improve both PFS^17^, ^18^, ^19^ and OS^6^, ^7^ in HR+/HER2− MBC. Drug use in the real world has patient population differences, especially age, and adjuvant hormonal therapy, compared with clinical trials. It is essential to study the impact of treatments on various age groups, as older patients may have different physiological characteristics, comorbidities, and treatment tolerances. As an example, the patient age in this study was higher than PALOMA‐2 and PALOMA‐3.^18^, ^19^ In this study, patients over 65 years old accounted for 61.8%, while in PALOMA‐2 and PALOMA‐3 40.8% and 25.0%, respectively. The result drawn from this study is that CDK4/6i has a similar effect in older patients with ABC compared to younger patients, and their use does not result in an increased rate of AE. Interestingly, the MONALEESA‐7 study explored the efficacy and safety of ribociclib plus ET in premenopausal women with advanced, HR+ breast cancer and found that ribociclib plus ET can improve PFS compared with ET and has a manageable safety profile.^20^ In this real‐world study, eight premenopausal women were treated with ribociclib plus ET for ABC. We found that ribociclib plus ET had a therapeutic response (CR: 1 case, PR: 4 cases). Therefore, the number of premenopausal women treated with ribociclib are expected to increase in the future and more evidence of real‐world drug use is still needed to provide a reference for clinical treatment.

Palbociclib and ribociclib are currently approved for HR+/HER2− MBC based on PALOMA‐2, MONALEESA‐2, and MONALEESA‐7 trials.^17^, ^18^, ^20^ To date, no randomized clinical trial has directly compared the efficacy and tolerability of palbociclib and ribociclib. In addition, patients in randomized trials are carefully selected populations in controlled settings and often do not reflect real‐world clinical practice. Therefore, real‐world data are urgently needed to better understand how these treatments are performed in unselected populations and to determine whether these drugs show similar benefits in terms of routine clinical practice. Based on the results of our real‐world data, there was no statistically significant difference in PFS between palbociclib and ribociclib treatments (Figure 6).

Our study had higher ORR and DCR than the pivotal studies of these drugs. The possible reason is that more patients (27/50; 54%) received first‐line ribociclib in our study. In addition, the combination of CDK4/6i plus ET is the most effective treatment for HR+/HER2− ABC. Nevertheless, the use of CDK4/6i in the neoadjuvant or metastatic setting has not been standardized. In our study, most patients (95.3%) were found to be combined with CDK4/6i plus ET, which showed a tendency to improve PFS after Cox regression multivariable analysis, but there was no significant difference. Some previous studies have shown that combining CDK4/6i plus ET can improve the treatment response rate of patients.^21^, ^22^ However, some studies have shown no significant improvement in clinical response rates.^23^, ^24^ The sample size of both these and our studies is small. In future, a comprehensive analysis of this topic should be conducted to obtain more summary results.

In addition, some real‐world studies have explored the efficacy and safety of CDK4/6i. The study by Edman Kessler et al. showed that the efficacy of CDK4/6i was lower than the results of RCTs, but still had a similar safety endpoint. From these results the authors believe real‐world use of CDK4/6i is more for patients after chemotherapy, thus resulting in markedly reduced efficacy.^25^ However, the retrospective study by García‐Trevijano et al. evaluated the efficacy and safety of 66 patients using CDK4/6i.^26^ The study analyzed the therapeutic effects and AE of palbociclib and ribociclib, respectively, and the results showed that the median PFS of palbociclib was 12.76 months and ribociclib was not reached. As for the toxicity profile, neutropenia was the most common AE, and they found that the occurrence of neutropenia could be controlled by changing the treatment regimen. However, there was a higher median PFS, ORR, and DCR in our study than real‐world studies, and the possible reason for this was that CDK4/6i was used less in patients after chemotherapy in this study, thus resulting in greater efficacy.

This study had some limitations. First, it was a retrospective single‐center study, and some information could not be found in the electronic medical records; therefore, it was prone to information bias. Second, the sample size was small which may have led to chance findings or masked correlations, affecting the results. Despite these limitations, this real‐world study of ribociclib and palbociclib, with a long follow‐up time, provides an insight into the real‐world use of CDK4/6i.

In conclusion, drug treatment should be based on the accumulation of new evidence and treatment experience and the treatment guidelines should be adjusted accordingly, especially the drugs for cancer. It is expected that in the future, the treatment of breast cancer should be based on the patient's status, disease status, previous treatment response, whether to combine other drugs, safety, economics, and personal preferences. Thus, studies of drug use in real‐world settings provide new evidence for future clinical practice.

## AUTHOR CONTRIBUTIONS

Conception and design: TRP.

Drafting of the article: TRP, TWW.

Literature search: TRP, JHC, TWW.

Figures: TRP.

Data collection: TRP, JHC, TWW.

Data analysis and statistical expertise: TRP.

Obtaining funding: TRP, TWW.

Supervision the article: JHC, TWW.

All authors approved the final version of the manuscript. Transparency: The lead authors affirm that the manuscript is an honest, accurate, and transparent account of the study being reported; that no important aspects of the study have been omitted; and that any discrepancies from the study as planned have been explained.

## CONFLICT OF INTEREST STATEMENT

No potential conflict of interest relevant to this article is reported.
